# Role of Radio Telescopes in Space Debris Monitoring: Current Insights and Future Directions

**DOI:** 10.3390/s25092900

**Published:** 2025-05-04

**Authors:** Bhaskar Ahuja, Luca Gentile, Ajeet Kumar, Marco Martorella

**Affiliations:** 1 Department of Physics, University of Trento, Via Sommarive 14, 38123 Trento, Italy; bhaskar.ahuja@unitn.it; 2 RaSS National Laboratory, National Inter-University Consortium for Telecommunications (CNIT), 56124 Pisa, Italy; luca.gentile@cnit.it (L.G.); m.martorella@bham.ac.uk (M.M.); 3 Microwave Integrated Systems Laboratory (MISL), University of Birmingham, Birmingham B15 2TT, UK

**Keywords:** radio telescope, bistatic radar, debris tracking, space surveillance and tracking, passive radar

## Abstract

The growing population of space debris poses significant risks to operational satellites and future space missions, necessitating innovative and efficient tracking solutions. Ground-based radar for space surveillance has been a central area of research since the early Space Age, with recent advancements emphasizing the use of bistatic radar systems that incorporate sensitive radio telescopes as receivers. This approach offers a cost-effective and scalable solution for monitoring space debris. Preliminary observations demonstrated the viability of employing radio telescopes in bistatic configurations for effective debris tracking. This review provides a comprehensive analysis of experiments utilizing radio telescopes as bistatic receivers, highlighting key advancements, challenges, and potential applications in space surveillance systems. By detailing the progress in this field, this study underscores the critical role of bistatic radar systems in mitigating the growing threat of space debris.

## 1. Introduction

### 1.1. Background and Motivation

Space debris presents several significant risks to space operations and long-term sustainability of space activities [1]. Collision hazards are a primary concern, as debris can damage or destroy operational satellites, spacecraft, and the International Space Station (ISS). The risk of Kessler Syndrome, in which debris collisions generate more debris, threatens the viability of certain orbital regions, particularly the Low Earth Orbit (LEO) [2]. The presence of space debris also increases the operational costs for space agencies and satellite operators, necessitating investment in tracking systems, collision avoidance maneuvers, and protective shielding. Ultimately, the increasing quantity of debris threatens the long-term sustainability of space activities, presenting a complex challenge for the global space community. As part of Space Situational Awareness (SSA) [3], Space Surveillance and Tracking (SST) focuses on technologies such as radar, optical telescopes, and advanced data analysis algorithms to identify and forecast the paths of active satellites and space debris with precision [4]. A global network of surveillance sites is continuously being developed to ensure uninterrupted space monitoring. The United States maintains a comprehensive catalog through its Space Surveillance Network (SSN), utilizing both optical and radar sensors [5,6]. Based on the data collected by these sensors, Two-Line Element (TLE) sets are generated and made freely available to promote international cooperation, enabling orbit propagation using the standard SGP4 algorithm [7].

Radar has been employed in space surveillance studies since the early years of the Space Age because of its key benefit of operating efficiently under all weather conditions without the need for sunlight, which optical sensors do [8]. In addition to traditional radar systems, complementary techniques such as phased array radars and LIDAR (Light Detection and Ranging) have been employed for tracking smaller space debris due to their enhanced accuracy and rapid beam steering [9,10]. However, the high cost of phased arrays and the technical challenges associated with high-power lasers in LIDAR systems limit their scalability [11,12]. Thus, research is increasingly focusing on developing innovative and cost-effective solutions for space surveillance by leveraging existing infrastructure and passive sensing techniques.

**Figure 1:**
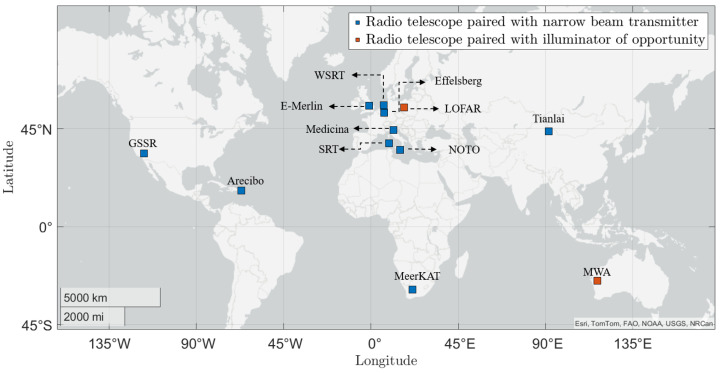
Locations of radio telescopes around the world involved in past space debris monitoring campaigns. Blue squares represent systems used with active narrow beam transmitters, while red square indicate passive systems that utilize illuminators of opportunity as transmitters (generated using MATLAB R2024a, www.mathworks.com; accessed on 6 March 2025).

In particular, systems that employ a sensitive radio telescope as a bistatic receiver operating in beam-park mode have gained interest, due to their potential for wide-area coverage with reduced infrastructure requirements [13]. Radio telescopes, originally designed for observing faint cosmic signals [14], have demonstrated their capability as receivers for radar applications. Researchers have been investigating the use of radio telescopes as radar receivers for space surveillance applications, and several experiments have been performed with configurations of interest. A few of these experiments, with a special focus on the LEO region, were reported in [15]; however, an extensive review is still missing. This article comprehensively examines debris monitoring experiments conducted using radio telescopes in the past, provides a detailed analysis, and elucidates the progress made to date in this field. Figure 1 shows the locations of radio telescopes around the globe that have been utilized in past space monitoring campaigns. Several countries, including Australia and South Africa, have contributed to such efforts; however, Europe remains the primary contributor, particularly in advancing innovative radio-telescope-based approaches. Furthermore, the classification of systems into active and passive types, based on their transmitter configuration, is explained in the following section.

### 1.2. Structure of the Review

The aim of this section is to clarify the boundaries of the review and explain how the content is organized. Firstly, the suitability of radio telescopes for space debris monitoring, along with their advantages, is discussed in Section 2. Secondly, based on the available literature, the radio telescope experiments have been categorized into two main state-of-the-art configurations, classified by the type of transmitter involved.

**Figure 2:**
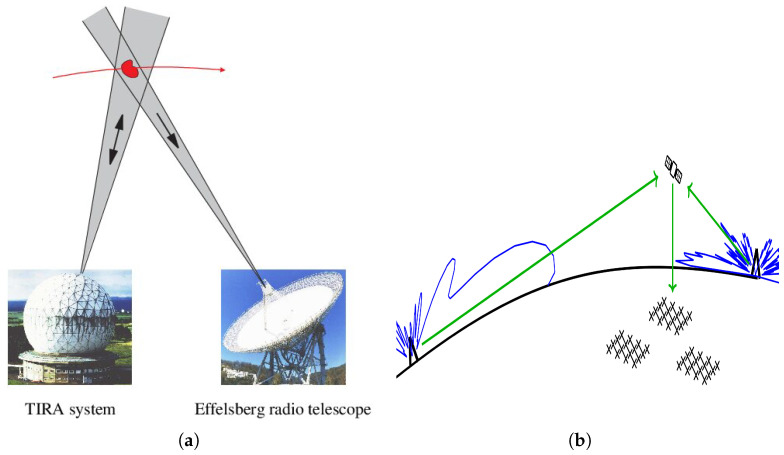
Types of configurations based on the nature of the transmitter used: (**a**) An example of a narrow-beam active transmitter system: TIRA used as the transmitter and Effelsberg as the receiver, reproduced from [16]. (**b**) An example of a passive configuration using illuminators of opportunity: Murchison Widefield Array (MWA) operating as a receiver with external transmitters, reproduced from [17].

- Active systems are those in which the radio telescope acts as a receiver in conjunction with a separate high-power narrow-beam radar transmitter. These systems emit controlled signals and analyze the reflections from space objects. However, the narrow beams of the radar and receiver limit the coverage area. This is consistent with the operation of most space-surveillance radar systems that use single narrow beams or fence configurations. One example of this kind of system is the Effelsberg radio telescope with Tracking and Imaging Radar (TIRA) as the transmitter [16]. Figure 2a illustrates an active bistatic configuration of the TIRA–Effelsberg system, where a narrow beam transmitter TIRA illuminates the target and the Effelsberg radio telescope captures the reflected signal.
- Passive bistatic radar systems rely on existing sources of illumination, such as FM and DVB signals. Unlike active radar systems, passive radar does not require a dedicated transmitter, making it the most cost-effective alternative. In this setup, single or distributed arrays of radio telescopes are paired with broadband transmitters located at significant distances. The wider field of view of the receiver, combined with the broad broadcast coverage of the transmitter, results in a significantly larger surveillance volume. Although this setup decreases the power concentration on space objects, it offers the benefit of a longer detection time for space debris, allowing precise orbital calculations without requiring previous data. Moreover, a distributed ground broadcast network allows several simultaneous sources of illumination. One such example is the Murchison Widefield Array (MWA) in Australia [17]. Figure 2b illustrates a passive radar configuration, where MWA functions as a receiving system capturing signals reflected from a space object, using non-cooperative transmitters (illuminators of opportunity).

**Figure 3:**
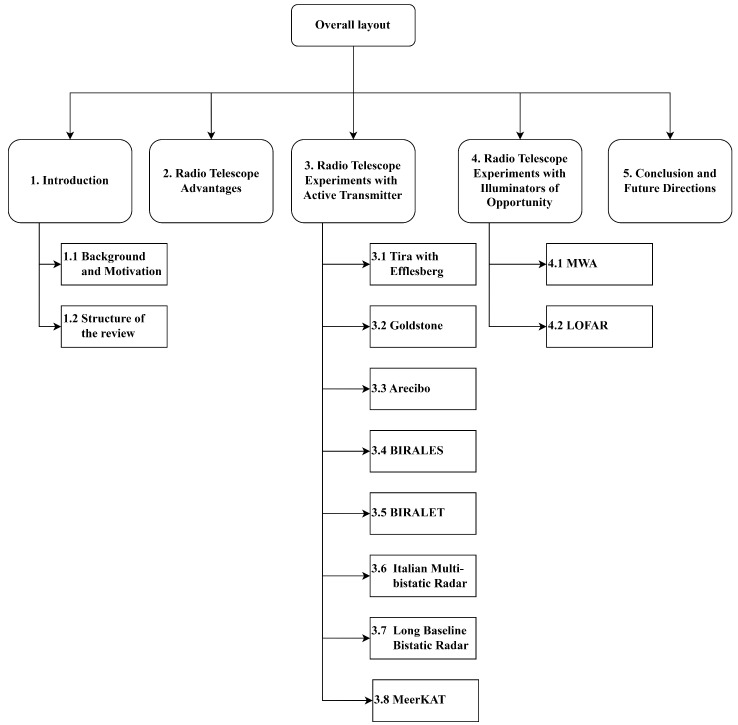
This flowchart presents the overall layout of the review article, including classification of radio telescope experiments based on the type of transmitter used and the radio telescopes involved in the past experiments.

The structure of the following sections reflects this division, with separate discussions on experiments with active transmitters and passive setups using illuminators of opportunity. Section 3 reviews experiments conducted with narrow beam transmitters, and Section 4 then discusses experiments carried out involving illuminators of opportunity. Figure 3 illustrates the structure of the review with classification of radio telescope experiments based on the type of transmitter used and lists the notable telescopes involved in experiments conducted under each configuration. Lastly, the conclusions and discussion are presented in the final section of the article. In summary, this article discusses the outcomes of the experiments conducted and presents a broad overview of the current state of research, along with a discussion of future scope.

## 2. Why Use Radio Telescopes?

One of the main reasons behind using radio telescopes as receivers is their capability to detect small debris that could not be observed by traditional monostatic radars. This can be understood by the well-known radar Equation in (1) ([18] pp. 1.11–1.13).

(1) $$SNR=\frac{P_{t}G_{t}G_{r}\lambda^{2}\sigma }{{\left(4\pi \right)}^{2}R_{t}^{2}R_{r}^{2}LkT_{sys}B}$$

where $P_{t}$ denotes transmitted power. $G_{t}$, $G_{r}$ are the transmitter and receiver gains, λ is the wavelength, σ is the radar cross-section (RCS) of the debris. $R_{t}$, $R_{r}$ are the range from the target to the transmitter and receiver, respectively, *k* is Boltzmann’s constant, $T_{sys}$ represents the system noise temperature, *B* represents the bandwidth of the system, and SNR represents the signal-to-noise ratio. The benefits of using a radio telescope as a bistatic receiver are summarized as follows:

1. The high sensitivity of radio telescopes arises from their large collecting areas. According to antenna theory, receiver antenna gain ($G_{r}$) in Equation (1) can be expressed as
  (2) $$G_{r}=\frac{4\pi A_{eff}}{\lambda^{2}}$$
  where $A_{eff}$ denotes the effective reception area. The larger the effective area, the larger the signal-to-noise ratio. Hence, the large aperture size of the radio telescopes significantly enhances their detection range.
2. The lower system noise temperature ($T_{sys}$) of radio telescopes is another key factor contributing to their higher SNR, as it is inversely proportional. This improved sensitivity is achieved by cryogenically cooling the receiver front-end, including the low-noise amplifiers, to temperatures as low as 4 Kelvin using liquid helium. Such cryogenic cooling significantly reduces thermal noise and enhances the overall sensitivity of the system [19].
3. In a bistatic configuration, separating the transmitter and receiver enhances angular diversity, minimizing blind spots and improving detection capabilities. The total bistatic range, given by $R_{b}=R_{t}+R_{r}$, provides greater flexibility in observation geometry. This configuration complements traditional monostatic radar systems by enhancing coverage and redundancy [20].
4. Radio telescopes can also contribute to debris characterization by analyzing radar cross-sections σ at multiple scattering angles, providing detailed information about the size, shape, and material composition of debris, as σ is a function of $f(\theta_{inc},\theta_{sca},\phi ,\lambda )$, where $\theta_{inc}$ is the incident angle, $\theta_{sca}$ is the scattering angle, and ϕ is the polarization. This detailed profiling is vital for understanding the physical properties of debris and assessing its potential threats [21].
5. Interferometric capabilities from modern phased array radio telescopes such as the Square Kilometre Array (SKA) enable high-resolution imaging of space debris, allowing precise visualization of debris clouds and their spatial distribution.
6. Using these radio telescopes in a multistatic configuration becomes beneficial, as the systems already work in synchronization for Very Long Baseline Interferometry (VLBI) or similar projects.
7. Another benefit of using a radio telescope is to utilize the existing infrastructure, primarily designed for other purposes, which makes the overall system cost-effective.

These features, coupled with the ability to leverage the existing infrastructure, make radio telescopes indispensable tools for enhancing SSA.

## 3. Radio Telescope Measurements with Active Transmitters

Previous investigations have shown that several available facilities around the globe participated in collaborative space debris monitoring, based on the first architecture discussed in Section 1.2. These measurement campaigns have included Germany’s Effelsberg Radio Telescope in collaboration with TIRA since 1996 [8], which is described in detail in Section 3.1. Another validation experiment was performed in the late 90s using Goldstone bistatic Radar in the USA [22], which is discussed in Section 3.2. The Arecibo Radio telescope, once the largest telescope in the world, in Puerto Rico, reported a few debris observations during the early 1990s. Sadly, this architecture has been dismantled due to collapse in 2020, as discussed in Section 3.3 [23]. Moreover, an initial stage space debris observational test was conducted in 2007 using the Medicina–Evpatoria bistatic radar system, where the system consisted of the Evpatoria RT-70 radar antenna in Ukraine as the transmitter and the Medicina VLBI 32 m dish in Italy as the receiver [24]. Unfortunately, due to the ongoing war in Ukraine, the Evpatoria antenna is no longer available for collaboration with the Western world.

Moreover, Italy has two active bistatic radars based on radio telescopes. One of them is the BIRALES system, whose acronym stands for BI-static RAdar for LEO Survey, located in Medicina, Bologna [25]. The other is the BIRALET system, which is an acronym for BI-static RAdar for LEO Tracking, and its receiver is the Sardinia Radio Telescope (SRT) located in Sardinia [26]. A detailed review and the results from the BIRALES and BIRALET experimental campaigns are discussed in Section 3.4 and Section 3.5, respectively. Our group in Pisa, Italy, has also contributed to this field by investigating the performance metrics and operational benefits of multibistatic configurations using three Italian radio telescopes as receivers [27,28,29], and it is discussed in detail in Section 3.6. In the last few years, experiments have been conducted by NATO Science and Technology Organization (STO) members for Geostationary Earth Orbit (GEO) monitoring [30], and it is reported in Section 3.7. A relevant additional investigation was carried out with SKA in South Africa, specifically utilizing the MeerKAT radar system, which is also explored in detail in Section 3.8.

Recent studies have further advanced the application of radio astronomy to space debris tracking. For instance, Tianlai radio arrays have been explored as part of a bistatic radar system for LEO debris detection [31]. Additionally, ref. [32] demonstrated that passive RF observations using collocated antennas can refine orbit determination and reduce TLE-based errors to below 100 m and 0.2 m/s, as validated by the Canadian Space Agency. However, these specific findings are beyond the scope of this article and have not been reported here.

### 3.1. TIRA with the Effelsberg Telescope

The Fraunhofer Institute for High-Frequency Physics and Radar Techniques (FHR), Germany, operates one of the largest radars in Europe, the Tracking and Imaging Radar (TIRA). It is capable of operating in both the L-band (1.33 GHz) as a narrow-band mono-pulse tracking radar and the Ku-band (16.7 GHz) as a high-range-resolution imaging radar [33]. In 1996, a space debris observation experiment was conducted using a bistatic radar formed with the TIRA system as the radar transmitter and the Effelsberg radio telescope as the radar receiver [8]. The Effelsberg radio telescope has a 100 m dish antenna and is operated by the Max Planck Institute for Radio Astronomy in Germany. Several experiments using TIRA as the transmitter/receiver and the Effelsberg radio telescope as a secondary receiver to lower the detection threshold have been performed since 1996 [34,35,36]. Figure 2a illustrates the geometry of the experiment, showing the TIRA transmitter and the Effelsberg radio telescope as the receiver.

**Figure 4:**
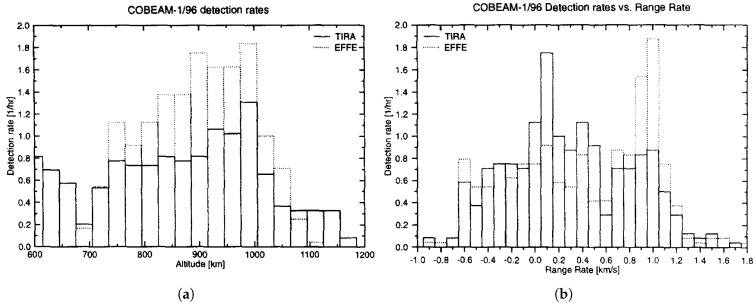
Results from the first COBEAM experiment performed in 1996: (**a**) Detection rate (detections per hour) versus altitude, shown in 30 km bins, comparing performance of the TIRA L-band radar and the Effelsberg radio telescope. Effelsberg demonstrates significantly higher detection rates across all altitude ranges due to its superior sensitivity. (**b**) Detection rate versus range rate in 100 m/s bins over the interval −1 to 1.8 km/s. The TIRA detections peak around 0.1 km/s, indicating more detections of near-stationary or slowly moving objects, while Effelsberg shows a dominant detection rate near 1 km/s, corresponding to faster-moving targets in its field of view [34]. Reprinted from Advances in Space Research, Copyright 1999, with permission from Elsevier.

The initial COoperative BEAM-park mode (COBEAM) experiment was conducted over a 24-h period and successfully collected observational data on objects as small as 0.9 cm at a range of 1000 km [34]. Figure 4a illustrates the detection rate (detections per hour) against altitude (at 30 km intervals) for the TIRA L-band radar and Effelsberg. Effelsberg achieved a notably higher detection rate owing to its higher sensitivity, even though its beam width was only one-third that of the TIRA L-band beam. Figure 4b displays the detection rates compared with range rates over the −1 to 1.8 km/s limits, segmented into 100 m/s increments. The distribution peaks at 0.1 km/s for data from TIRA, whereas the Effelsberg data predominate around 1 km/s. Following the first promising results of the COBEAM experiment, researchers have refined estimation and calibration procedures to further enhance system performance [37]. The authors in [38] showed a moderate improvement in the detection capability after revising the multidimensional maximum likelihood estimation for determining object trajectories and RCS between beam park campaigns in 2008 and 2009. Ongoing TIRA upgrades, including Ka-band polarimetric imaging and L-band tracking enhancements, will significantly boost space surveillance and support future bistatic measurement campaigns [33]. Expanding on the successful bistatic radar experiments involving TIRA-Effelsberg, which improved the detection of space debris in low Earth orbit, the German Experimental Space Surveillance and Tracking Radar (GESTRA) is set to further enhance these capabilities. Its phased-array design inherently supports bistatic and multistatic configurations, leading to improved detection accuracy and estimation performance [9].

### 3.2. Goldstone Orbital Debris Radar

**Figure 5:**
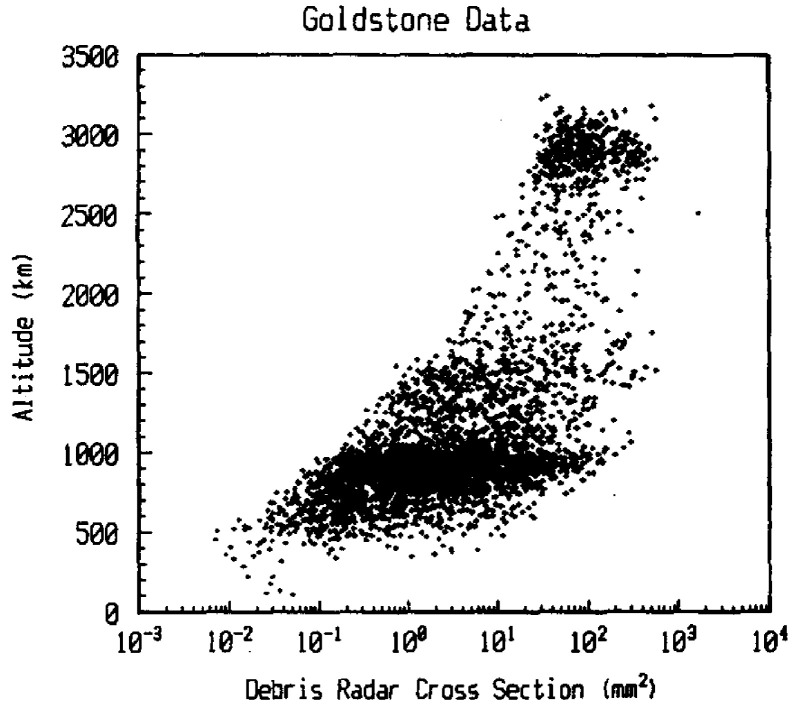
Radar cross-section (RCS) versus altitude of targets detected by the Goldstone bistatic radar during a 3-year period (1994–1997). The plot highlights the distribution of object sizes and altitudes, reflecting the radar’s ability to detect a range of space objects across different orbital regions [22]. Reprinted from Advances in Space Research, Copyright 1999, with permission from Elsevier.

The Goldstone Orbital Debris Radar operated by NASA, is a highly capable system used for monitoring the orbital debris environment. It has supported multiple space debris observation campaigns [39]. Between October 1994 and May 1997, the Goldstone Solar System Radar, together with a 35-m parabolic antenna acting as the receiver, operated in beam-park mode. During this period, approximately 110 h of observations were conducted, resulting in the detection of 3476 objects [22]. Figure 5 presents a summary of this measurement campaign, where the minimum radar cross-section (RCS) of the detected objects is plotted against their corresponding altitudes. A particularly noteworthy finding from this study was the unexpected identification of a concentration of debris around 2900 km in altitude, a group not previously observed by other radar systems. Additionally, very few objects were detected below 300 km, which the authors attributed to the geometric limitations of the intersecting radar beam [22]. In a related study, Stokley et al. [40] compared debris flux measurements from three radar systems: the Goldstone Radar, Haystack Imaging Radar, and Haystack Auxiliary Radar (HAX) before the year 1998, during 2001, and after 2003, the last solar maximum. Furthermore, the findings from the measurement campaigns conducted between 2016 and 2017 are reported here [41]. Thanks to several upgrades in bandwidth and the integration of dual-polarization capabilities over the last few years, the detection efficiency of the system has improved significantly, enabling the detection of debris as small as 3 mm.

### 3.3. Arecibo Observatory

**Figure 6:**
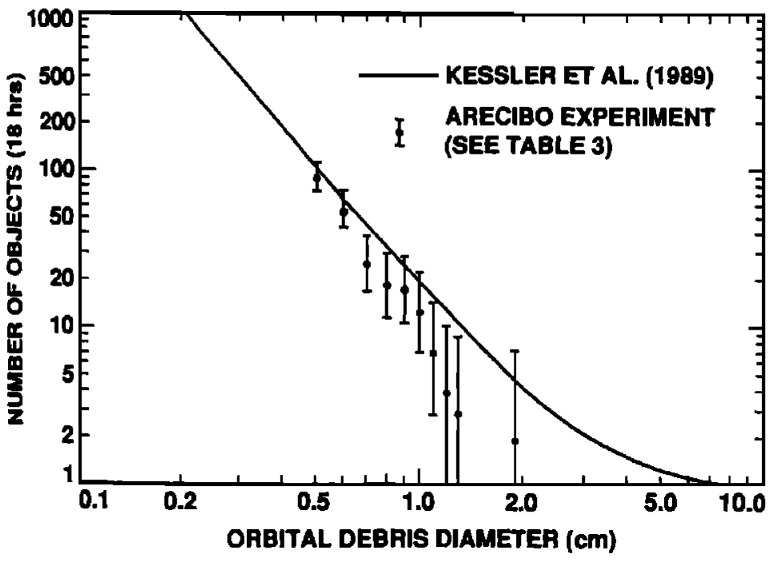
Comparison results from the Arecibo debris radar experiment (June–July 1989), showing detected objects plotted against diameter. Around 19 h of data collection led to the detection of 90 objects between 5 mm and 20 mm [23]. Reprinted from Geophysical Research Letters, Copyright 1992, with permission from John Wiley and Sons.

The Arecibo Observatory was once among the world’s largest and most sensitive radio telescopes. During a debris radar experiment conducted from late June to early July 1989, the Arecibo Observatory collected approximately 19 h of data and detected 90 objects ranging in size from 5 mm to 20 mm in diameter [23]. The experiment utilized the Arecibo radar as the transmitting antenna, while a 3.5 m reflector auxiliary antenna, positioned near the main observatory, served as the receiving antenna. These antennas were separated by a 10 km baseline and were aligned to observe a common point at an altitude of 575 km, and to detect the smallest objects, ranging from 0.5 to 2 cm, by comparing the observed results with a predictive model. The comparison results from the experiment are shown in Figure 6, where the detected objects are plotted against their diameter. Unfortunately, the recent structural failure of the Arecibo dish has resulted in the loss of these significant capabilities. However, the proposal for a Next Generation Arecibo Telescope (NGAT) holds promise for improved radar performance compared with the already substantial legacy system. This enhanced sensitivity could be leveraged to address the knowledge gaps in the orbital debris environment within the size range of 1 mm to 1 cm in low Earth orbit, while simultaneously obtaining measurements across an underrepresented range of orbital inclinations [42].

### 3.4. BIRALES

**Figure 7:**
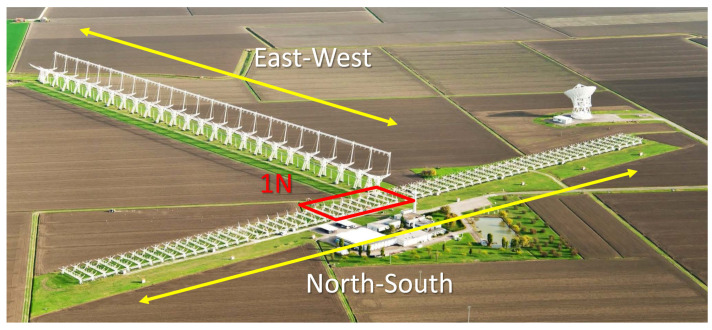
BIRALES receiving antenna, part of the Northern Cross radio telescope located at the Medicina Observatory in Bologna, Italy. It consists of eight parabolic cylindrical antennas arranged along the north—south arm depicted in red box, with a total collection area of approximately 1400 m^2^ [44]. Reprinted from Acta Astronautica, Copyright 2020, with permission from Elsevier.

Both the BIRALES and BIRALET systems utilize a common transmitter, the Radio Frequency Transmitter (TRF), which consists of a 7 m dish wheel-and-track steerable parabolic antenna. The transmitter is situated in the Italian Joint Test Range within the region of Cagliari, Sardinia, Italy. TRF is capable of generating a peak power of 10 kW and operates with a 410–415 MHz bandwidth [43]. The receiving antenna is part of the Northern Cross radio telescope, which is currently one of the largest UHF-capable antennas worldwide [44], as shown in Figure 7. The components of the BIRALES receiver include eight parabolic cylindrical antennas located along the north–south (N-S) arm with a combined collection area of approximately 1400 square meters, enabling the detection of objects as small as 10 cm at a slant range of 2000 km [45]. BIRALES has achieved notable success in tracking debris owing to its higher sensitivity and strategic location in central Italy. The current 32-receiver multibeam configuration of BIRALES allows the angular track of the space object to be calculated to complement the bistatic range and Doppler measurements [46,47]. This innovative technique outperforms conventional single-beam radar because when an object passes through the field of view of the radar, it is illuminated by radio waves, and the beam illumination sequence helps define the angular trajectory of the target, as explained in [46].

BIRALES began its measurement campaigns with initial trials in 2014, during which the bistatic radar successfully detected around 20 objects at altitudes between 300 and 850 km. A comprehensive summary of the experimental measurements performed by BIRALES in 2014 is reported in the comprehensive analysis by Muntoni et al. [15]. BIRALES is designed to simultaneously receive Continuous Wave (CW) and chirp signals from the TRF for Doppler and range measurements, respectively [48]. With the help of the multibeam approach, the time profiles of the range, Doppler, and angular position can be used to perform initial orbit determination (IOD) for unknown objects and reconstruct the track [49]. Consequently, an IOD algorithm was developed, particularly according to BIRALES system measurements, to tackle the problem of element spacing longer than half the wavelength [44]. Researchers have reported a range accuracy of 50 m and an angular accuracy of $10^{-3}$ deg, which were utilized by the orbit determination block to perform statistical orbit determination using measurement data from BIRALES during single- or multipass observations [50]. In 2018, BIRALES participated in tracking the re-entry of Tiangong-1 with observations conducted on 29 and 31 March. The re-entry time was predicted by propagating the state estimates, with the 31 March observation aligning well within the European Space Agency’s forecasted window [51].

Another study involving BIRALES radar observation and reconstruction of the Cosmos 1408 fragmentation event, which occurred on 15 November 2021, following an anti-satellite missile test, was conducted by a team from Politecnico di Milano, the Italian Space Agency, and the Italian National Institute of Astrophysics [52]. In a recent article [53], results from the third Long March 5 B re-entry campaign, which took place from 31 October to 4 November 2022, were derived. BIRALES observations were combined with measurements from the MFDR-MR sensor, a tracking radar involved in the campaign and managed by the Italian Air Force. Orbit determination results were computed to obtain a re-entry epoch prediction, and a novel method for estimating the ballistic coefficient in a stochastic manner, which can be used in orbit determination and re-entry analysis, was also discussed by the authors. The predicted re-entry epoch was found to be consistent with the reference provided by the U.S. Space Command. A unique orbit determination pipeline was considered for the two-sensor scenario. Using the first set of observations from BIRALES, researchers calculated the initial orbit determination (IOD) with an adaptive beamforming approach [47]. Subsequently, predictions were made using an Unscented Kalman Filter (UKF) to propagate to the next observation set at the MFDR-MR tracking radar [54]. At the end of the measurements at MFDR-MR, the states were propagated again to the BIRALES sensor to obtain a second set of measurements to perform the refined orbit determination again. Table 1 presents the Keplerian parameters of the orbit determination results obtained, along with the reference parameters derived from the most recently available TLE. Note that BIRALES 1 and BIRALES 2 correspond to the first and second sets of measurements, respectively, and a(m), *e*, *i*, Ω, ω, and ν represent the semimajor axis, eccentricity, inclination, longitude of the ascending node, argument of periapsis, and true anomaly, respectively.

Overall, BIRALES demonstrates notable efficiency in detecting small objects owing to its large receiving area and real-time radar data processing backends [55]. This enables it to identify items just a few centimeters in size while significantly conserving energy. Future plans include upgrading 64 parabolic cylindrical reflectors on the N-S arm, totaling 256 receivers and over 10,000 m^2^ of the collecting area, and a new transmitting antenna for observation flexibility of any kind [56]. Maximum sensitivity is achieved when all reflectors are aligned in the same direction, enabling BIRALES to detect debris a few centimeters in size in LEO. This configuration reduces the field of view from 90° × 6.6° to 5.7° × 6.6° compared with independent cylinder pointing. This mode will be effective in specific cases, such as monitoring fragment clouds after satellite explosions and to prevent collisions with operational satellites.

### 3.5. BIRALET

BIRALET operates in the P-band at 410–415 MHz, has a bistatic configuration composed of the same transmitting antenna used in BIRALES and SRT as the receiver, with a baseline of approximately 20 km [13]. The SRT is a 64-m fully steerable wheel-and-track antenna located near San Basilio (Cagliari, Sardinia, Italy). In the initial phase, SRT participated in the debris monitoring campaign in 2014 jointly with the Northern Cross radio telescope and was able to detect six objects, as listed in Table 2. The authors described three possible hardware configurations of the system to perform Doppler shift and range measurements [25]. The first configuration utilizes the spectrum analyzer as a backend that permits Doppler shift measurements, whereas the second configuration is based on the electronic Red Pitaya board. The final configuration uses the National Instruments USRP board as a back end. The hardware has been constantly upgraded to perform space debris monitoring, including the re-entry of the Chinese space station Tiangong-1 together with other Italian SST sensors in 2018 [57,58].

**Figure 8:**
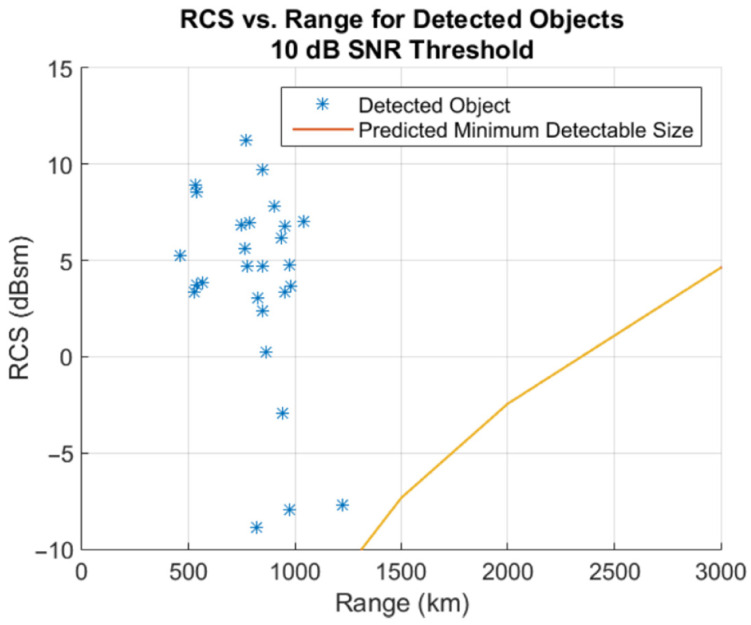
Scatter plot of radar cross-section (RCS) versus range for the detected objects, with reference to the minimum detectable size of the BIRALET system. Reproduced from [26].

Between 13 December 2018 and 10 October 2019, the BIRALET system was able to observe a total of 33 objects in the beam-parking mode using the dedicated back-end. The estimated and measured Doppler shifts for the detected debris between 13 December 2018 and 8 October 2019, can be found in the following literature [25,26]. Figure 8 shows a scatter plot of the RCS versus the range for the detected objects with respect to the minimum detectable size using the BIRALET system. These measurement campaigns serve as a solid foundation for monitoring space debris using radio telescopes.

### 3.6. Italian Multibistatic Radar

**Figure 9:**
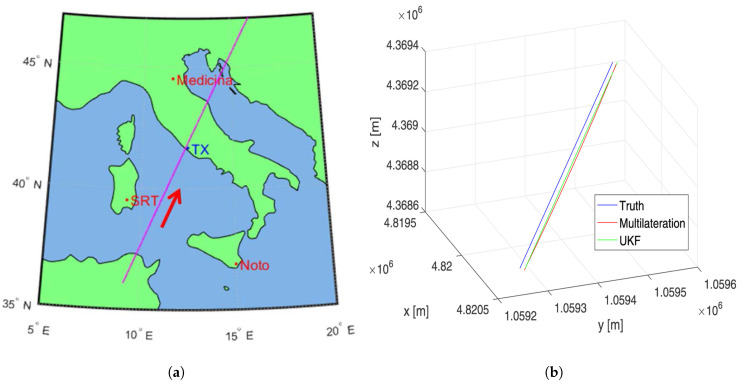
(**a**) Ground track of the target as it passes over the Italian radio telescopes—Medicina, NOTO, and SRT. (**b**) Three-dimensional global tracking results of the target’s truth path (blue), IOD-multilateration path (red), and UKF tracking path (green), demonstrating tracking accuracy and deviations [27]. Reprinted with permission from IEEE Aerospace and Electronic Systems Magazine, Copyright 2020, under license from IEEE.

A multibistatic radar system consists of multiple spatially separated antennas, typically with one serving as the transmitter and several others as receivers, or vice versa. The spatial diversity provided by such configurations enhances detection, tracking, and IOD capabilities. In recent years, the Radar and Surveillance Systems National Laboratory in Pisa initiated simulation-based studies on multibistatic radar systems. Cataldo et al. [27] proposed a configuration involving three Italian radio telescopes: Medicina, NOTO, and SRT. The Medicina observatory is the same one that is used in the BIRALES experiment, while SRT is the receiver in BIRALET [48]. The third radio telescope, NOTO, located in Sicily, is operated by the Istituto di Radioastronomia di Bologna. A simulation tool was developed to evaluate system performance for SSA missions under various scenarios. To manage system complexity, the authors proposed decentralized processing with track fusion, and addressed synchronization challenges using GPS-based clocks [28]. Different signal processing techniques were implemented to extract range and Doppler measurements by combining data from multiple sensors. Consequently, IOD was performed using a multilateration technique, while orbit tracking was carried out using an Unscented Kalman Filter (UKF). Additionally, the system was designed to associate newly detected tracks or correlate them with existing ones. Figure 9a shows the target ground track on a flat Earth crossing over considered sensors, and Figure 9b presents the tracking results in Earth-Centered Earth-Fixed (ECEF) coordinates, comparing the true trajectory (blue), multilateration output (red), and UKF-based estimation (green). In a recent study [59], the same research group demonstrated that the proposed multibistatic configuration can significantly enhance tracking performance. Their findings underscore the potential of this architecture to support not only space situational awareness (SSA) but also mission planning and navigation for low-thrust transfer trajectories.

Interestingly, these three Italian radio telescopes (Medicina, NOTO, and SRT), along with the Effelsberg radio telescope in Germany, have demonstrated the capability to detect Near-Earth Objects (NEOs) in a collaborative experiment with NASA [60]. This effort was part of a European Space Agency (ESA) project aimed at defining functional requirements for a future planetary radar system and assessing the suitability of existing European infrastructure for NEO observations. In parallel, the Planetary Radio Interferometry and Doppler Experiment (PRIDE), also implemented in ESA’s JUICE mission, represents a sophisticated evolution of multibistatic radar concepts [61]. PRIDE uses VLBI by coordinating globally distributed radio telescopes to derive high-precision spacecraft state vectors. Although originally developed for deep-space and planetary science applications, the PRIDE framework presents a promising approach for long-baseline, passive multibistatic tracking of high-altitude targets, such as GEO satellites. Although NEO and planetary surveillance are important applications of SSA, they lie outside the scope of this paper. Its potential for future extension to space debris monitoring is particularly relevant, as similar concepts inspire the long-baseline GEO surveillance approach discussed in the following section.

### 3.7. Long Baseline Bistatic Radar (LBBR)

The study, conducted by the NATO STO Research Task Group (RTG), investigated the use of existing monostatic radars as transmitters in combination with large-aperture radio telescopes as bistatic receivers for GEO monitoring [30,62]. This approach aimed to enhance the sensitivity of object detection at GEO distances. Monitoring geostationary satellites poses significant challenges due to their considerable distance of approximately 36,000 km from Earth. Addressing these challenges requires high-power radars, typically in the megawatt (MW) range, as well as international collaboration to establish the necessary bistatic configuration for effective detection and tracking of objects at GEO altitudes. Table 3 lists high-power radars that are either currently used for GEO monitoring or have the capability to track GEO targets due to their substantial transmission power. Only radars that can be configured in a bistatic setup with radio telescopes are included. Other high-power phased array radars, such as the Cobra Dane phased array radar with a peak power of 15.4 MW, are not listed here. However, the full list can be found in [15].

The experiments conducted by RTG involved two transmitting radars: the Millstone Hill Radar (MHR) in the USA and TIRA in Germany. These transmitters were paired with multiple receiving radio telescopes in Europe, including SRT in Italy, Westerbork Synthesis Radio Telescope (WSRT) in the Netherlands, and multiple antennas of the e-MERLIN (enhanced MultiElement Remotely Linked Interferometer Network) array in Manchester, United Kingdom. The research team conducted several experiments between 2020 and 2021, targeting known geostationary satellites, to test the feasibility and performance of the bistatic radar setup. In such scenarios, a large-aperture radio telescope is the best option to increase the sensitivity of the system, especially when dealing with large-distance detection. Consequently, the results demonstrate successful continental baseline coherent bistatic radar collections of GEO targets. It should be noted that there was no synchronization between the transmitters and receivers, and the only available time synchronization was provided by GPS, which was presumed sufficient for these experiments.

**Figure 10:**
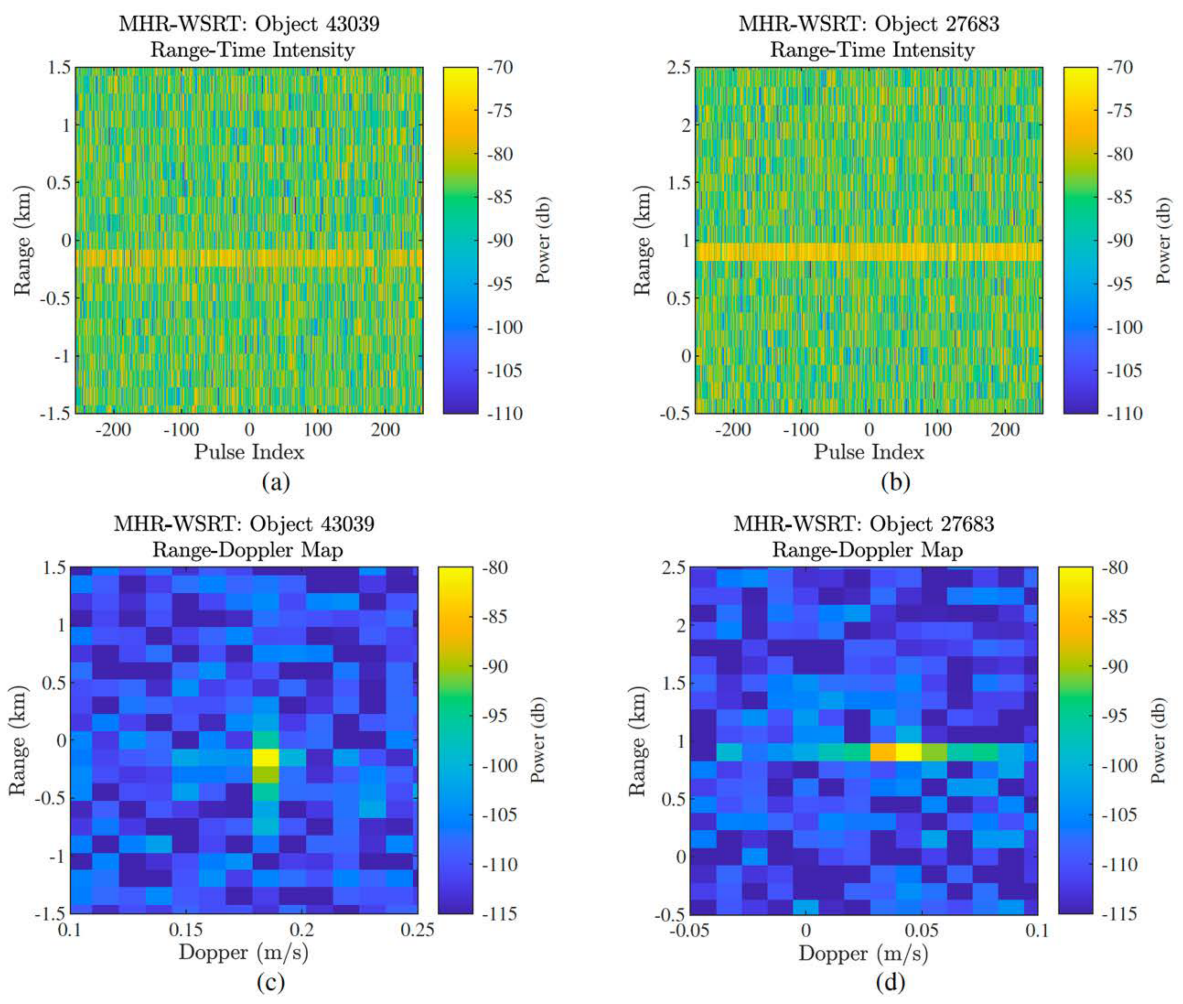
(**a**,**b**) Range—time intensity plots for objects 43039 and 27683, recorded during a single CPI in January 2020 using the MHR transmitter and WSRT receiver. These plots show signal strength variations over time and range. (**c**,**d**) Corresponding range–Doppler (RD) maps for the same targets. The origin (0,0) in each map is aligned with the expected range and Doppler shift of the respective object, aiding in accurate target identification and motion characterization [30].

**Figure 11:**
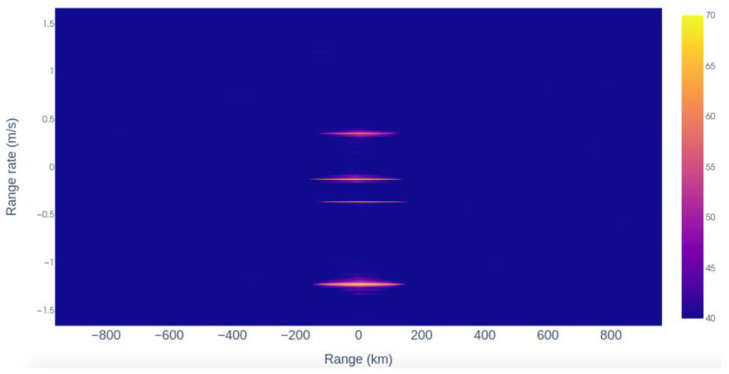
Range—Doppler map generated from the TIRA–Knockin bistatic radar data using a CPI of 512 pulses. The map clearly reveals four distinct targets detected during the January 2020 experimental campaign [62].

The team was able to perform range–Doppler (RD) processing after performing coherent integration for a long period and then determine the target RCS. The experimental outcomes for two targets (ID 43039 and 27683) in January 2020 using the MHR-WSRT combination are displayed in Figure 10. Specifically, Figure 10a,b present the range–time intensity plot, whereas Figure 10c,d show the RD maps for the same targets. It is important to note that the origin (0, 0) of the RD maps aligns with the expected target range and Doppler measurements. The bias in the range value plots could be a result of the timing difference between the transmit and receive site clocks and the TLE error, which can reach up to 25 km in the along-track direction and 10 km in the cross-track direction for GEO objects [63]. The observed Doppler offset may have resulted from an unmatched bistatic Doppler during pulse compression, leading to a residual phase ramp across the pulses. The research team also provided measurement results from the monostatic radar TIRA and bistatic results combined with the e-MERLIN radio telescope. During this measurement campaign, three antennas (Knockin: 25 m, Pickmere: 25 m, and Cambridge: 32 m) of the e-MERLIN array simultaneously received the TIRA signal at 1.33 GHz in both polarizations. RD analysis was performed, and four targets were detected using all three antennas, as can be seen in Figure 11. The study also highlighted the challenges and complexities of long-baseline bistatic radar, including timing synchronization, phase coherence, and the need for an accurate target ephemeris. To the best of our knowledge, orbit determination results are not included in the study.

**Figure 12:**
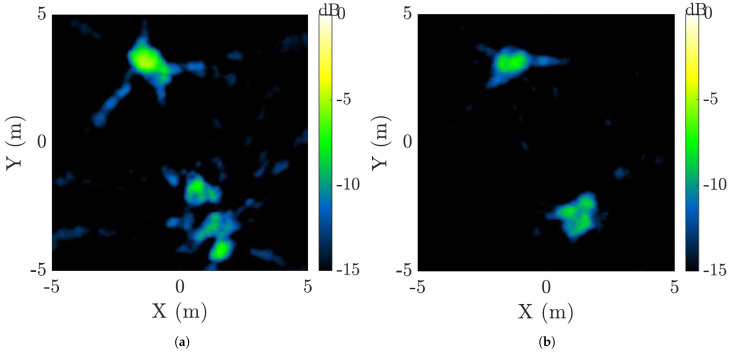
Bistatic Doppler tomography reconstructions of the Atlas 5 Centaur rocket body using L-band data with 8 MHz bandwidth, collected by WSRT. The results illustrate the target structure based on Doppler information: (**a**) PP—prime polarization channel, (**b**) OP—orthogonal polarization channel [64]. Reprinted from IET Radar, Sonar, and Navigation, Copyright 2023, with permission from John Wiley and Sons.

The same RTG performed monostatic and bistatic radar imaging experiments on real on-orbit tumbling rocket bodies by utilizing the same network of transmitters and receivers [64,65,66,67]. These studies demonstrated advanced bistatic Doppler characterization across diverse imaging geometries and highlighted the successful implementation of specialized Doppler processing techniques, such as the Doppler superpulse algorithm. The study in [64] also outlines the methodology for accurately determining the rotation periods and maximum observed length extent of tumbling objects in space. Figure 12 displays the bistatic Doppler tomography reconstructions for the Atlas 5 Centaur rocket body in the L-band with an 8 MHz bandwidth based on WSRT data collection. The PP and OP channels are shown in Figure 12a,b, respectively. Table 4 compares the rotation period estimates for Delta-4 and Atlas 5, derived from multiple receivers and different processing methods. Recent studies have presented a novel range–Doppler–time (RDT) tensor processing technique for GEO characterization with narrow-band radar, which provides detailed Doppler information [68].

In another study, Riofrío et al. [69] discussed the performance analysis of ground-based long baseline radar for SSA by examining targets at different altitudes. The study examines the performance of a multistatic system considering one transmitter in three scenarios: a cluster of receivers, receivers spread throughout the world, and a combination of both. The authors used the multiple-input–multiple-output (MIMO) ambiguity function to analyze the system’s performance, and simulations were performed for targets in LEO and GEO to check the feasibility of the proposed radar systems.

### 3.8. MeerKAT

**Figure 13:**
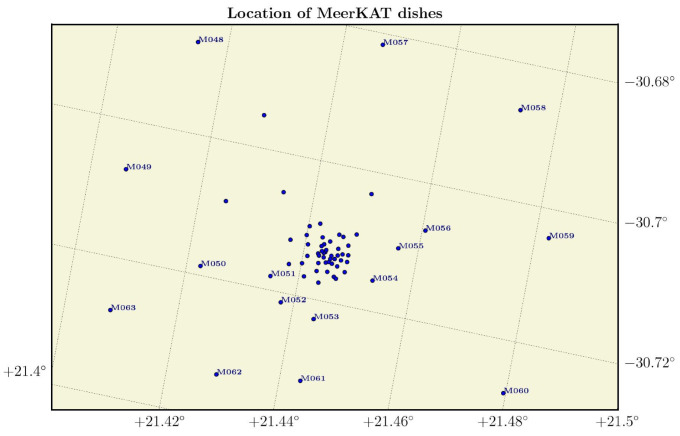
Map showing the layout of the MeerKAT radio telescope array. The receptors, labeled M000 to M063, are distributed across the site based on a two-dimensional Gaussian configuration [70].

The Meer Karoo Array Telescope (MeerKAT), located in the Northern Cape of South Africa, is part of the SKA network. It comprises 64 dishes intended for radio astronomy and serves as proof of technological advancement toward constructing a Square Kilometre Array (SKA), as shown in Figure 13. The MeerKAT radar is designed to operate at 1350 MHz (L-Band) frequency with a bandwidth of 10 MHz. The Radar Remote Sensing Group (RRSG) at the University of Cape Town, South Africa, laid the essential groundwork with simulations for the Mission Planning Tool (MPT), which will be used at the MeerKAT radar to perform sensor scheduling and orbit determination analysis [70,71]. Agaba’s PhD thesis [71] primarily concentrates on designing the comprehensive system operation and the radar signal processing for the proposed MeerKAT radar with the RRSG’s Flexible Extensible Radar Simulator (FERS) [72]. In addition, different radar configurations, such as monostatic, quasi-monostatic, and single-input–multiple-output (SIMO), were analyzed to determine the best performance with MeerKAT. The results indicate that the multistatic radar configuration is the most appropriate and computationally efficient option compared with the bistatic and SIMO configurations. The research presented in this PhD thesis also focused on Inverse Synthetic Aperture Radar (ISAR) imaging, comparing simulated results with theoretical models while developing methods to correct range and Doppler ambiguities, thereby improving imaging precision [71].

Moreover, in another study, a bistatic system was proposed with a transmitter located at Denel Dynamics’ Overberg Test Range in Arniston, Bredasdorp (Western Cape, South Africa) and a receiver at MeerKAT, separated by a baseline of approximately 450 km. A preliminary case study was conducted to demonstrate the operation of a bistatic passive radar system to observe the International Space Station [73]. The authors compared the capabilities of the MeerKAT radar to the BIRALES system, as described in Section 3.4, to estimate azimuth and elevation angles [70]. This comparison was necessary because the current design of MeerKAT does not include an angle of arrival estimation scheme. Nonetheless, the simulation results demonstrate the potential effectiveness of the MeerKAT radar in tracking LEO space debris. However, this must be validated through real-world measurement campaigns.

## 4. Radio Telescope Measurements with Illuminators of Opportunity

Typically, terrestrial broadcasting networks, including television and radio, comprise a distributed array of multiple transmitters. This configuration enables the simultaneous utilization of several transmitters as radar illumination sources above the receiver. Transmitting antennas are typically oriented towards the ground to maximize the power directed at the intended terrestrial targets. However, this design choice can present challenges for illuminating satellites, as considerable effort has been made to minimize outward radiation, with the main elevation sidelobes exhibiting power levels that are substantially lower, up to 15 dB, compared with the main beam [74]. Currently, there are at least two prominent facilities, the Murchison Widefield Array (MWA) located in Australia and part of the LOFAR telescope in Poland, which are actively working on developing and utilizing passive radar systems for space debris monitoring. These passive radar configurations leverage existing sources of illumination, such as FM and DVB signals, to detect and track space debris, thereby providing a cost-effective and expandable solution for this growing challenge. The MWA, like the MeerKAT radar, is part of the SKA project, and a detailed discussion is provided in Section 4.1. Measurement results from the LOFAR telescope are presented and discussed in detail in Section 4.2.

### 4.1. Murchison Widefield Array

**Figure 14:**
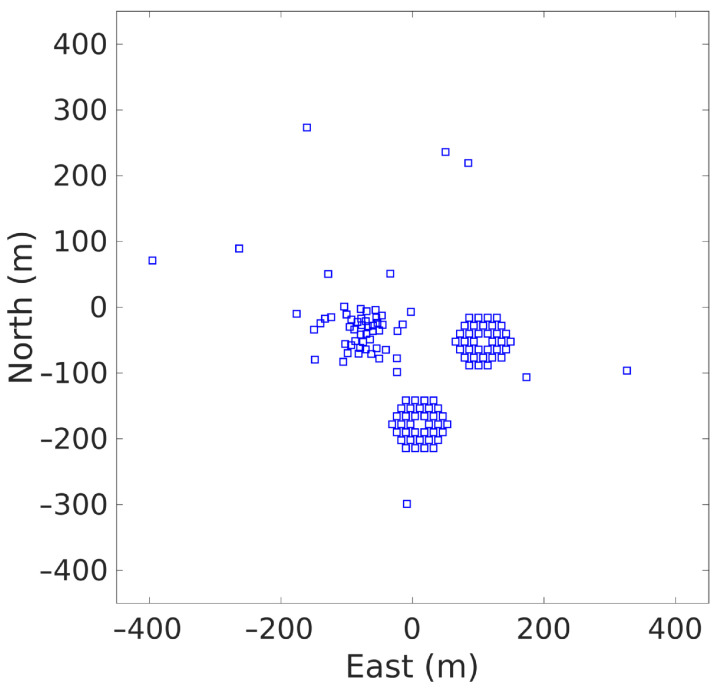
Layout of the Murchison Widefield Array (MWA). Each receiver is represented by a blue marker. The array consists of multiple tiles spread across the site to form a large interferometric baseline. Reproduced from [17].

The Murchison Widefield Array (MWA), a member of the trio of SKA Precursor telescopes, is situated at the Murchison Radio-astronomy Observatory in the Murchison Shire, located in the mid-west of Western Australia. This site was specifically chosen because of its exceptionally low radio-frequency interference. Operating in the low radio frequency range of 80–300 MHz, the MWA features a processed bandwidth of 30.72 MHz per linear polarization. It comprises 128 aperture arrays or tiles spread across an area with a diameter of approximately 3 km, as illustrated in Figure 14 [17,75]. The primary objectives of MWA include detecting neutral hydrogen from the epoch of re-ionization, investigating Earth’s ionosphere, and creating maps of both galactic and extragalactic radio sources. Earlier demonstrations used MWA to detect ISS and the moon using reflected FM radio emissions, but observations were limited to angular domain (i.e., right ascension and declination) only [76,77]. The experimental campaign conducted in April 2015 used both the MWA and a Defense Science and Technology group line-of-sight receiver in Adelaide. The primary target was the ISS, which was illuminated by nearby FM radio transmissions, as described in the scenario outlined in [78]. Researchers in [78,79] developed a passive radar signal processing technique to derive the bistatic range and Doppler shift with a coherent processing interval of 1 s. This technique improved the detection performance by 25–40 dB compared with the previous case where only angle observations were possible [80]. Additionally, azimuth and elevation measurements were derived through interferometric processing. These four measurements were combined to perform IOD and tracking by using the Markov chain Monte Carlo algorithm.

Hennessy et al. [81] introduced the concept of Orbit Determination Before Detect (ODBD) for MWA, which constrains the search space to realistic orbital trajectories and improves detection efficiency. This algorithm incorporates orbital parameters into the radar ambiguity function and applies matched filtering to single-beam radar systems. The authors further validated the algorithm by applying it to a real measurement campaign at MWA conducted in 2018 and comparing it with the standard IOD method, such as Herrick-Gibbs, demonstrating the practical implementation of the algorithm, which further enhances the accuracy of debris detection [82]. While the authors proposed ways to constrain the search space, it may still require significant processing power for practical implementation.

**Figure 15:**
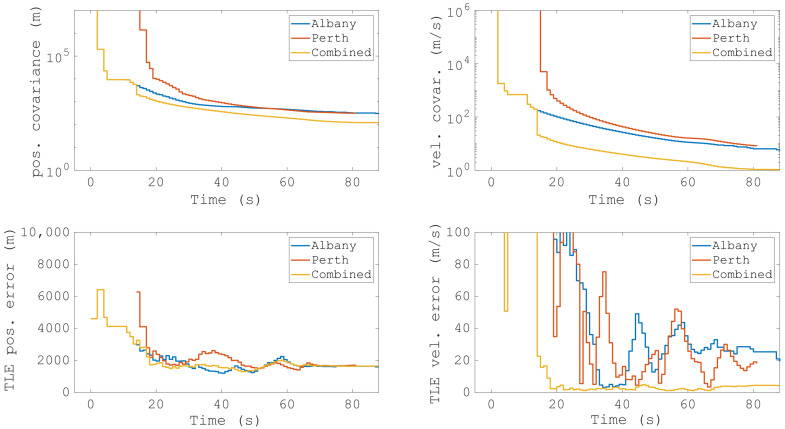
The resulting orbit predictions from the multistatic measurements of COSMOS 1707, conducted in December 2019 with the MWA radio telescope. The top rows show the covariance of the position estimate and the velocity estimate, and the bottom row shows the mean errors when compared with the Two-Line Element (TLE). Reproduced from [17].

Another measurement campaign was conducted in December 2019 using four transmitters (Geraldton, Perth, Albany, Mount Gambier) [17], and one of the detected targets, COSMOS 1707, was tracked for almost 90 s. The tracking results from the multistatic measurements are shown in Figure 15, with the top row as the covariance of position and velocity, and the bottom row shows the error estimates of position and velocity in comparison with the TLE ephemeris. The authors also mentioned that these results are typical for most of the objects detected at the MWA in a bistatic configuration. The measurements were combined at the orbit determination stage, and it can be seen in Figure 15 that the joint result from the Perth and Albany transmitters shows significant improvement in both position and velocity covariance. The mean error values, especially in the velocity, were reduced, which shows that the orbit accuracy improved with the combined data. In the same campaign, the tracking results of other targets, OPS 5721 (9415) and NADEZHDA (25567), are also published [17].

Moreover, a rigorous assessment was conducted between 2019 and 2023 by investigators to extract precise angular measurements of satellites from spatially blurred detections produced by non-coherent passive radar techniques using the MWA [83,84]. By developing specialized signal processing methods, researchers were able to derive time-stamped angular coordinates for 32 observed satellite passes, which were then used to perform high-accuracy orbit determination through a least-squares fitting algorithm. The resulting median uncertainties of 860 m in the cross-track and 780 m in the in-track directions have been reported for LEO objects, which are comparable to the typical 1000 m uncertainty in publicly available TLE data [85,86]. Over the past decade, the development of MWA has been successful, with significant improvements in passive radar signal processing techniques that have enhanced the ability to refine the orbit determination of space objects. Advancements in MWA in this area have contributed to more accurate tracking and monitoring of the growing population of space debris, which poses escalating risks to operational satellites and future space missions.

### 4.2. LOFAR

The Netherlands Institute for Radio Astronomy, ASTRON, developed and built the LOw-Frequency ARray for radio astronomy (LOFAR), an international network of radio telescopes. LOFAR began operations in 2012 and currently comprises 52 independent stations. Of these, ASTRON manages 38 stations, whereas the remaining 14 are international stations situated across Europe [87,88]. Earlier research conducted by a group of researchers at the Warsaw University of Technology (Poland) explored the application of LOFAR radio telescopes as passive radar receivers [89]. Initially, the focus was on the possibility of detecting airplanes, but the research was expanded to include the observation of space objects traveling in LEO. The research team carried out experimental trials to validate the concept, utilizing a LOFAR station as the receiver and commercial digital audio broadcasting (DAB) transmitters as illuminators of opportunity for aerial object detection [90,91].

**Figure 16:**
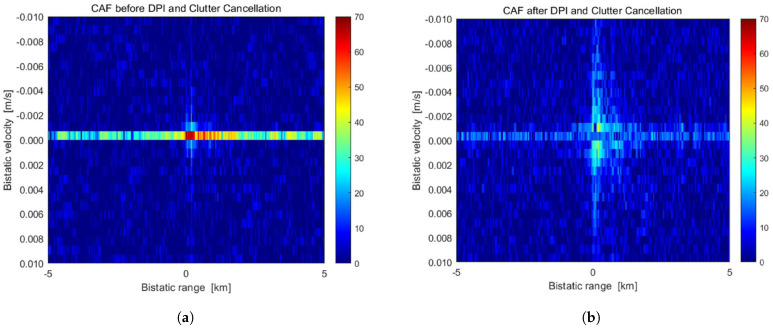
(**a**) A zoom on cross -ambiguity function (CAF) near-zero bistatic range and velocity before direct-path interference (DPI) and clutter cancellation. (**b**) A zoom on cross-ambiguity function (CAF) near zero bistatic range and velocity after direct-path interference (DPI) and clutter cancellation, showing improved target signal clarity in the bistatic domain. The data were obtained using the LOFAR PL610 station with a DVB-T transmitter as an illuminator of opportunity for ISS detection [92]. Reprinted from IET Radar, Sonar, and Navigation, Copyright 2023, with permission from John Wiley and Sons.

In a previous study, researchers employed the LOFAR station PL610 in Borówiec, Poland, and a proximate DVB-T transmitter to detect ISS [92,93]. The utilization of DVB-T offers the advantage of providing a broader bandwidth and potentially more precise range measurements compared with previously utilized DAB+ transmitters [93]. The cross-ambiguity function (CAF) calculated in [92] is displayed in Figure 16, showing its appearance both prior to and following the implementation of Direct Path Interference (DPI) and clutter cancellation techniques. Figure 16a clearly shows the presence of DPI and clutter elements. In contrast, Figure 16b illustrates the successful removal of these interferences, demonstrating the efficacy of the cancellation method. This enhancement results in a clearer CAF, which allows for a more precise identification of target signals in the vicinity of the zero bistatic range and velocity. This enabled the detection of weak signals from distant objects. The successful detection of ISS across a wide range highlights the capability of passive radar systems for space surveillance [94,95]. Recent studies have further demonstrated the potential of this approach by detecting Starlink satellites [96], which have significantly lower radar cross-sections than the ISS, showcasing the feasibility of exploiting the existing radio astronomy infrastructure to enhance space situational awareness.

## 5. Conclusions and Future Directions

An extensive review of past space debris monitoring campaigns using radio telescopes is conducted in this study. The challenges of monitoring space debris are compounded by the need to track fragments as small as 5 mm, as even at this size, they can cause significant damage to active satellites. Globally, more than 50 radar systems are currently dedicated to monitoring space objects and debris, and efforts are underway to expand this network of radar systems designed for space surveillance. Among these, bistatic radars with radio telescopes provide a balanced approach that combines high sensitivity and cost-effectiveness for space debris monitoring. In this study, an important perspective is explored by organizing the debris monitoring campaigns into two segments based on the type of transmitter used. A comparative summary of the radio telescopes discussed in this study is available in Table 5, listing relevant attributes including location, setup, transmission features, and detection performance. The analysis presented in Table 5 and the review of existing literature reveal that more campaigns have been conducted with active transmitter configurations, likely due to the advantage of higher transmitted power. Nevertheless, opportunistic transmitters are also favored because of their all-time availability and long exposure durations. This study included measurement campaigns for both LEO and GEO orbital regions, but most of the campaigns were conducted to monitor the LEO orbit, as it is highly populated with space debris. However, monitoring GEO targets is particularly challenging because of their large distance from the Earth, and it involves coordinating multiple countries to establish the necessary bistatic configuration and requires high transmission power, as emphasized in Section 3.7. Some of the radio telescopes discussed also operate in a multistatic configuration, which, despite introducing additional system complexity, significantly enhances overall sensitivity. This improvement, along with the transition from bistatic to multistatic configurations, provides advantages in terms of precise localization and measurement ambiguity reduction, as highlighted in Section 3.6, Section 3.7 and Section 4.1 (indicated by ^†^ in Table 5).

There are a few limitations associated with conventional radio telescopes for space debris monitoring, and they have been highlighted in this manuscript. A common issue encountered when using radio telescopes is obtaining precise angular measurements, as most radio telescopes provide only range and Doppler measurements. Determining the orbit of an uncataloged object using only these two types of measurement can be challenging. However, some of the radio telescopes discussed here have addressed this problem through unique designs such as multibeam antennas and advanced data processing algorithms, as detailed in Section 3.4 and Section 4.1, respectively. Single-beam radio telescopes still face difficulties in deriving accurate angular measurements, which could be an area of future research and improvement.

Several studies [11,97,98,99] suggested that integrating space-based radar and radio telescopes with existing space situational awareness programs can significantly improve debris tracking and collision avoidance strategies. This approach can enhance the detection accuracy, expand coverage beyond the low Earth orbit, and enable real-time monitoring of hazardous space debris. On the other hand, some of the radio telescopes discussed in this paper, such as BIRALES, MeerKAT, MWA, and LOFAR, operate based on the Very Long Baseline Interferometry (VLBI) principle or similar interferometric techniques, which are known to enhance spatial resolution and measurement accuracy. In addition, future research can expand the characterization of space objects using multistatic interferometric ISAR imaging, as very limited research has been conducted in this domain. A notable application of high-resolution radar imaging, utilizing similar principles, is the IoSiS experiment conducted by the German Space Agency, DLR, for imaging satellites and space debris in LEO [100,101]. Moreover, the mathematical model proposed in the recent literature [102] for the 3D imaging of resident space objects based on the interferometric approach needs to be further validated using real-world multistatic receiver networks. Its independence from the sensor system geometry makes it a promising approach for improving space object tracking and identification. This direction is further supported by the PRIDE experiment discussed in Section 3.6, which applied interferometric techniques for deep-space tracking and may inspire similar methodologies for space debris imaging in high-altitude orbits.

The comprehensive analysis in this manuscript reveals that space situational awareness efforts are primarily led by the USA and European countries, while relatively fewer experiments have been conducted in other parts of the world. Research institutions and space agencies in the Asia-Pacific region, such as ISRO and JAXA, have the potential to adopt innovative approaches and further advance this technology. It should be emphasized that one of the key reasons for the limited analysis reported so far is the lack of publicly available data, as some space agencies classify such work for security and strategic reasons. Although the USA and Europe have been significant contributors, engaging more stakeholders from other parts of the world could further propel progress in this domain. In addition to government initiatives, a few private companies are also contributing to space situational awareness, such as LeoLabs from the USA and Silentium Defense from Australia [103,104]. While LeoLabs uses phase-array technology to precisely track targets in space, the Maverick radar from Silentium Defense utilizes passive FM signals to detect and determine the orbit of debris. Moreover, advances in computational power and the refinement of machine learning and deep learning techniques have driven increasing interest in applying these methods to improve orbit prediction accuracy and resident space object classification [105,106,107,108]. Ongoing advancements in radar and radio telescope systems, along with improvements in data analysis and machine learning methods, are crucial for advancing future space debris tracking. By addressing existing technical issues and promoting international collaboration, researchers can devise more effective and scalable strategies to mitigate the increasing threats posed by space debris accumulation.

## Figures and Tables

**Table 1:** Keplerian parameters for the Orbit determination results obtained on 4 November from BIRALES and MFDR observations, along with parameters computed from the last available TLE [53]. Reprinted from Advances in Space Research, Copyright 2025, with permission from Elsevier.

	*a* [km]	*e*	*i* [deg]	Ω [deg]	ω [deg]
BIRALES 1	6530.3	0.020	41.5	77.4	179.6
MFDR-MR	6533.6	0.003	41.6	82.3	158.2
BIRALES 2	6517.4	0.004	41.6	82.1	172.6
Reference	6516.1	0.002	41.6	82.8	201.0

**Table 2:** Summary of the debris measurements by Bistatic Radar for LEO Tracking (BIRALET) in 2014, using the Sardinia Radio Telescope (SRT) as the receiver. During this campaign, six objects in LEO were successfully detected. Reproduced from [13].

Object ID	Name	Doppler (Hz)	Altitude (km)	Slant Range (km)	RCS (m^2^)
22565	COSMOS 2237 (First passage)	not measured	853.6	1732	11.6
22565	COSMOS 2237 (Second passage)	−5200	852.7	2836	11.6
33320	HJ-1A (First passage)	6200	628.7	3640	1.5
33320	HJ-1A (Second passage)	4600	629.3	1832	1.5
32783	CARTOSAT 2A	5200	629.4	2056	2.3
13552	COSMOS 1408	not measured	523.7	1093	8.4
16206	COSMOS 1375	3400	984.8	2326	0.5
38047	VESSELSAT 2	5200	460.7	1664	0.3

**Table 3:** List of high-power radar transmitters with the capability to detect GEO targets. The table includes radar systems that are either currently used for GEO monitoring or are suitable for such applications.

Name	Country of Origin	Peak Power	Operating Frequency
TIRA	Germany	1.6 MW	1.33 GHz
Millstone Hill Radar	USA	3 MW	1.295 GHz
Haystack Radar	USA	400 kW	10 GHz
Goldstone Radar	USA	440 kW	8.56 GHz
EISCAT * UHF Radar	Sweden	2 MW	930 MHz
EISCAT Svalbard Radar (ESR)	Norway	1 MW	500 MHz

* European Incoherent Scatter Scientific Association.

**Table 4:** The table shows the results of rotation period estimation using different methods: **TAC**—time–domain auto-correlation, **TFAC**—time–frequency domain auto-correlation, **STC**—slow-time cepstrum, **SFC**—slow frequency spectogram, **SIRTA**—spectrogram–inverse Radon transform-based algorithm [64]. Reprinted from IET Radar, Sonar, and Navigation, Copyright 2023, with permission from John Wiley and Sons.

Target	Receiver	TAC	TFAC	STC	SFS	SIRTA	Average
Delta-4	WSRT	164.2373	164.1280	164.2228	164.1748	164.4878	164.2501
	JBO-KN	164.0500	164.0257	164.0103	161.3476	164.6694	164.1889
	JBO-DE	164.2581	164.1588	164.2345	162.1518	164.3632	164.2537
	JBO-DA	164.2790	164.2472	164.3130	162.5722	164.9472	164.4466
	JBO-CM	164.1957	164.2717	164.3948	166.5494	164.5672	164.3573
Atlas 5	WSRT	33.6552	33.6567	33.6627	33.6651	33.6490	33.6577
	TIRA	33.6388	33.6507	33.6654	33.6221	34.8242	33.6442

JBO—Jodrell Bank observatory, KN—Knockin, DE—Defford, DA—Darnhall, CM—Cambridge.

**Table 5:** Overview of the radio telescopes analyzed in this study, providing a comparative summary of the systems examined.

Configuration	Sensors Involved (Tx/Rx)	Country	Configuration	Operating Frequency	Waveform Type	Detection Performance	References
TIRA with Effelsberg Radio Telescope	TIRA (Tx), Effelsberg (Rx)	Germany	Active	1.33 GHz	Pulsed	Targets ≥ 1 cm RCS detectable at 1000 km	[34]
Goldstone orbital debris radar	GSSR (Tx), DSS (Rx)	USA	Active	8.56 GHz	Pulsed	Targets ≥ 3 mm RCS detectable at 1000 km	[22]
Arecibo Observatory	Arecibo Telescope (Rx)	USA	Active	430 MHz	Pulsed	Targets ≥ 5 mm RCS detectable at 1000 km	[23]
BIRALES	TRF (Tx), Medicina (Rx)	Italy	Active	410–415 MHz	CW	Targets ≥ 1 m RCS detectable at 1000 Km	[51]
BIRALET	TRF (Tx), SRT (Rx)	Italy	Active	410–415 MHz	CW	Targets ≥ −8 dBsm RCS detectable at 1000 km	[26]
Italian Multibistatic Radar ^†^	Medicina (Rx), NOTO (Rx), SRT (Rx)	Italy	Active	-	-	N.A.	[27]
Long baseline bistatic radar ^†^	TIRA (Tx), MHR (Tx), WSRT (Rx), E-Merlin (Rx), SRT (Rx)	Germany, USA, Netherlands, UK, Italy	Active	1.333 GHz (TIRA), 1.295 GHz (MHR)	Pulsed	Detection at GEO range	[62]
MeerKAT ^†^	Denel Dynamics (Tx), MeerKAT (Rx)	South Africa	Interferometer-Active	1350 MHz	Pulsed	N.A.	[70,73]
Murchison Widefield Array (MWA) ^†^	MWA (Rx)	Australia	Interferometer—Passive	80–300 MHz	Passive (FM broadcast)	Targets ≥ 0.5 m^2^ RCS detectable at 1000 Km	[17]
LOFAR	LOFAR PL160 (Rx)	Poland	Interferometer—Passive	110–240 MHz	Passive (FM broadcast)	Targets ≥ 0.1 m^2^ RCS detectable	[91,92]

^†^ Multistatic-capable.

## Data Availability

Not applicable.

## Abbreviations

The following abbreviations are used in this manuscript:

ISS	International Space Station
LEO	Low Earth Orbit
SSA	Space Situational Awareness
SST	Space Surveillance and Tracking
SSN	Space Surveillance Network
TLE	Two Line Element
LIDAR	Light Detection and Ranging
TIRA	Tracking and Imaging Radar
MWA	Murchison Widefield Array
RCS	Radar Cross-Section
SKA	Square Kilometer Array
VLBI	Very Long Baseline Interferometry
BIRALES	Bistatic Radar for LEO Survey
BIRALET	Bistatic Radar for LEO Tracking
SRT	Sardinia Radio Telescope
STO	Science and Technology Organization
GEO	Geostationary Earth Orbit
GESTRA	German Experimental Space Surveillance and Tracking Radar
NGAT	Next Generation Arecibo Telescope
TRF	Radio Frequency Transmitter
IOD	Initial Orbit Determination
CW	Continuous Wave
UKF	Unscented Kalman Filter
ECEF	Earth Centred Earth Fixed
NEO	Near-Earth Objects
PRIDE	Planetary Radio Interferometry and Doppler Experiment
LBBR	Long Baseline Bistatic Radar
RTG	Research Task Group
MHR	Millstone Hill Radar
WSRT	Westerbork Synthesis Radio Telescope
E merlin	enhanced MultiElement Remotely Linked Interferometer Network
MIMO	Multi Input Multi Output
MeerKAT	Meer Karoo Array Telescope
RRSG	Radar Remote Sensing Group
MPT	Mission Planning Tool
FERS	Flexible Extensible Radar Simulator
SIMO	Single-Input–Multiple-Output
ISAR	Inverse Synthetic Aperture Radar
ODBD	Orbit Determination Before Detect
CAF	Cross-Ambiguity Function
DPI	Direct Path Interference
